# SwinDAF3D: Pyramid Swin Transformers with Deep Attentive Features for Automated Finger Joint Segmentation in 3D Ultrasound Images for Rheumatoid Arthritis Assessment

**DOI:** 10.3390/bioengineering12040390

**Published:** 2025-04-05

**Authors:** Jianwei Qiu, Grigorios M. Karageorgos, Xiaorui Peng, Soumya Ghose, Zhaoyuan Yang, Aaron Dentinger, Zhanpeng Xu, Janggun Jo, Siddarth Ragupathi, Guan Xu, Nada Abdulaziz, Girish Gandikota, Xueding Wang, David Mills

**Affiliations:** 1 GE HealthCare Technology & Innovation Center, Niskayuna, NY 12309, USA; grigoriosmarios.karageorgos@gehealthcare.com (G.M.K.); soumya.ghose@gehealthcare.com (S.G.); dentinge@gehealthcare.com (A.D.); millsda@gehealthcare.com (D.M.); 2 Department of Biomedical Engineering, University of Michigan, Ann Arbor, MI 48109, USA; xiaoruip@umich.edu (X.P.); zhanpeng@umich.edu (Z.X.); janggunj@med.umich.edu (J.J.); drsiddarthragupathi@gmail.com (S.R.); guanx@umich.edu (G.X.); xdwang@umich.edu (X.W.); 3 GE Vernova Research, Niskayuna, NY 12309, USA; zhaoyuan.yang@ge.com; 4 Division of Rheumatology, Department of Internal Medicine, University of Michigan, Ann Arbor, MI 48109, USA; nabdulaz@med.umich.edu; 5 Department of Radiology, University of North Carolina at Chapel Hill, Chapel Hill, NC 27599, USA; girish_gandikota@med.unc.edu

**Keywords:** rheumatoid arthritis, pyramid Swin transformers, automated 3D segmentation, deep attentive features, 3D ultrasound

## Abstract

Rheumatoid arthritis (RA) is a chronic autoimmune disease that can cause severe joint damage and functional impairment. Ultrasound imaging has shown promise in providing real-time assessment of synovium inflammation associated with the early stages of RA. Accurate segmentation of the synovium region and quantification of inflammation-specific imaging biomarkers are crucial for assessing and grading RA. However, automatic segmentation of the synovium in 3D ultrasound is challenging due to ambiguous boundaries, variability in synovium shape, and inhomogeneous intensity distribution. In this work, we introduce a novel network architecture, Swin Transformers with Deep Attentive Features for 3D segmentation (SwinDAF3D), which integrates Swin Transformers into a Deep Attentive Features framework. The developed architecture leverages the hierarchical structure and shifted windows of Swin Transformers to capture rich, multi-scale and attentive contextual information, improving the modeling of long-range dependencies and spatial hierarchies in 3D ultrasound images. In a six-fold cross-validation study with 3D ultrasound images of RA patients’ finger joints (*n* = 72), our SwinDAF3D model achieved the highest performance with a Dice Score (DSC) of 0.838 ± 0.013, an Intersection over Union (IoU) of 0.719 ± 0.019, and Surface Dice Score (SDSC) of 0.852 ± 0.020, compared to 3D UNet (DSC: 0.742 ± 0.025; IoU: 0.589 ± 0.031; SDSC: 0.661 ± 0.029), DAF3D (DSC: 0.813 ± 0.017; IoU: 0.689 ± 0.022; SDSC: 0.817 ± 0.013), Swin UNETR (DSC: 0.808 ± 0.025; IoU: 0.678 ± 0.032; SDSC: 0.822 ± 0.039), UNETR++ (DSC: 0.810 ± 0.014; IoU: 0.684 ± 0.018; SDSC: 0.829 ± 0.027) and TransUNet (DSC: 0.818 ± 0.013; IoU: 0.692 ± 0.017; SDSC: 0.815 ± 0.016) models. This ablation study demonstrates the effectiveness of combining a Swin Transformers feature pyramid with a deep attention mechanism, improving the segmentation accuracy of the synovium in 3D ultrasound. This advancement shows great promise in enabling more efficient and standardized RA screening using ultrasound imaging.

## 1. Introduction

Rheumatoid arthritis (RA) is an autoimmune condition, in which the immune system targets and damages the synovium which lines the joints. This immune reaction results in inflammation of the affected tissue, which may result in joint deformities, bone erosion, and functional impairment. RA impacts approximately 0.5–1% of the global population [1,2]. Early and accurate diagnosis of RA is essential for developing effective treatment strategies and mitigating complications [3,4].

Early RA can be identified using medical imaging modalities, including magnetic resonance imaging (MRI) and ultrasound (US) [5]. MRI has been shown to be capable of accurately detecting joint inflammation, compared with histological examination of synovial tissue [6,7]. However, MRI is limited by its availability, lengthy scan times, and cost [8]. US imaging shows potential in overcoming such limitations due to its capability for real-time visualization, affordability, portability and widespread accessibility. Power Doppler (PD) is commonly integrated with standard B-mode US imaging to map blood flow patterns in the affected joints and assess the degree of inflammation. However, US imaging involves limitations, including user dependency and variability among observers, posing challenges when it comes to establishing consistent diagnostic standards [8]. Recently, photoacoustic (PA) imaging has emerged as a promising modality for detecting RA. By integrating optical contrast with ultrasonic resolution, PA imaging exhibits high sensitivity to hemoglobin, which is the key biomarker in RA. This makes PA imaging highly appropriate for RA detection and has demonstrated potential in enhancing rheumatology diagnostics [9,10,11,12].

A critical part of assessing RA in US imaging is the process of isolating synovial tissue from B-mode images, which allows for the analysis of PA or PD blood flow measurements in the respective regions. The main challenges in synovium segmentation from US images include the complexity of interpreting the varying shades of gray in the images, the difficulty of accurately detecting bones due to noise and artifacts, and the need for precise localization of skin borders and joint regions [13]. These issues lead to false positives and inaccuracies in synovitis region identification, making the automation of this process particularly challenging. Manual segmentation heavily relies on the operator and can introduce high inter-observer variability.

**Figure 1:**
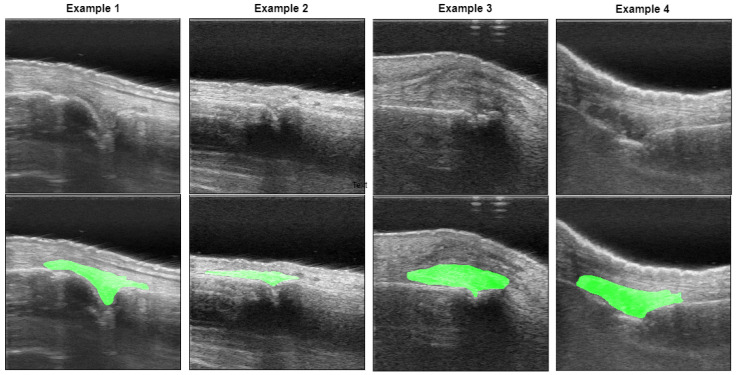
Examples of US images of the synovium with corresponding segmentation overlays (green). The images illustrate the challenges posed by ambiguous boundaries, large variations in synovium shape, and inhomogeneous intensity distribution.

To circumvent this shortcoming, automated segmentation approaches have been developed. A technique that uses confidence maps to automatically delineate the surface of the bone in hand joints in US scans has been previously proposed in [14]. Another approach for automated detection of multiple anatomical regions, including skin, bones and joints for RA grading in US, has been presented in [13]. Moreover, an approach for synovial tissue segmentation based on the active contour algorithm, combined with identification of skin border, bones, and joint center coordinates has been developed [15]. These existing methods focus on 2D US images, which present several challenges. Firstly, it requires more operator time and skill to position the probe in the optimal 2D plane for diagnostic-quality images of the finger joint. Furthermore, the PA signal may not be strong enough in the selected 2D plane, reducing the effectiveness of RA detection. Lastly, there can be high variability in the 2D plane selection due to the operator’s experience level. Therefore, 3D US images are more feasible, as they do not require the operator to have a high skill or experience level, and measurement variability can also be reduced. However, automatic segmentation of the synovium in 3D US remains challenging due to ambiguous boundaries, variability in synovium shape, and inhomogeneous intensity distribution (see Figure 1).

Recent advancements in deep learning (DL) have demonstrated significant promise in US image segmentation, markedly improving the accuracy and efficiency of identifying anatomical structures. DL algorithms leverage neural networks that can learn highly complex patterns of multiple structures, effectively addressing the specific challenges associated with US imaging, including inconsistencies in image quality and the presence of noise [16]. These algorithms have been successfully applied to various organs and anatomies, including the heart [17], lungs [18], liver [19], breast [20], blood vessels [21], kidneys [22], bones [23], nerves [24], and teeth [25], demonstrating great potential in diagnosing pathologies such as atherosclerosis, heart disease, liver fibrosis, kidney disease, RA, and various types of cancer.

Convolutional neural networks (CNNs) are highly effective DL models that have been widely utilized in the field of medical image segmentation. However, traditional CNN-based architectures, such as 3D UNet [26], tend to capture localized spatial information but may not effectively capture long-range dependencies and multi-scale contextual information, which are crucial for accurate 3D segmentation in US images. Recently, Swin Transformers [27,28] have been introduced as hierarchical vision transformers. Unlike conventional Vision Transformers (ViTs) [29], Swin Transformers partition images into non-overlapping patches and employ a hierarchical structure, facilitating efficient multi-scale feature extraction. At each hierarchical stage, Swin Transformer blocks process these patches at varying resolutions, effectively capturing both local and global contextual information. Moreover, the integration of a shifted window partitioning scheme significantly enhances the model’s capability to encode spatial dependencies by alternating the attention windows across layers, thus allowing interactions between adjacent non-overlapping windows [27,30]. These advancements have made Swin Transformers effective and adaptable for a wide range of downstream tasks, including medical image segmentation. Furthermore, Swin Transformers have demonstrated superior adaptability across different modalities of medical imaging [31,32,33,34]. Their hierarchical nature allows them to maintain computational efficiency while scaling to larger image resolutions, which is particularly beneficial for 3D US images where fine details and broader context must be simultaneously considered.

In this work, we introduce a novel network architecture, Swin Transformers with Deep Attentive Features for 3D segmentation (SwinDAF3D). This model builds upon the DAF3D architecture proposed by Wang et al. [35], by replacing the ResNeXt [36] backbone with a Swin Transformers Feature Pyramid Network (Swin FPN). The key novelties and contributions of our SwinDAF3D network include the following:

- Integration of Feature Pyramid with Swin Transformers: SwinDAF3D integrates hierarchical Swin Transformers enhanced by a shifted window mechanism into a Feature Pyramid, outperforming other transformer-based models such as Swin UNETR [31], UNETR++ [37], TransUNet [38].
- Utilization of Deep Attention Mechanism: SwinDAF3D uniquely integrates deep attention mechanisms with Swin FPN, allowing the model to focus on relevant features while suppressing irrelevant ones, thereby refining the overall feature extraction process in 3D US images.
- Clinical Impact for RA Assessment: SwinDAF3D improves automated synovium segmentation accuracy in 3D US images compared to baseline models, a critical advancement for reliable RA assessment and monitoring.

Furthermore, an ablation study is conducted to demonstrate that the developed SwinDAF3D model effectively leverages the combined strengths of Swin FPN and deep attention mechanisms. This study compares the segmentation performance of SwinDAF3D against five baseline models: 3D UNet [26], DAF3D [35], Swin UNETR [31], UNETR++ [37], and TransUNet [38], demonstrating improved performance in synovium segmentation for 3D US images. These advancements show great promise in enhancing diagnostic accuracy and improving clinical outcomes for patients with RA.

## 2. Materials and Methods

### 2.1. Data Acquisition

A total of *n* = 19 patients with active synovitis were recruited for this study. Patient screening and recruitment was carried out in collaboration with the Division of Radiology at the University of Michigan Hospital. Informed consent was obtained from all participants, and the study was approved by the University of Michigan’s institutional review board (IRB) (protocol no. HUM00003693). The patients’ finger joints were scanned using a GEHC VE95 US unit with an L8-18i-D high-frequency linear probe (GE HealthCare, Chicago, IL, USA). Real-time 3D US imaging was performed using a robotic hand scanning system, which can provide simultaneous and co-localized US and PA imaging, as described by Peng et al. [39]. The robotic hand captured multiple 2D B-mode US images in a stop–move–scan manner using a step size of 0.2 mm. The acquired 2D data were subsequently stacked together in order to obtain 3D US images. A total of *n* = 102 3D US images of the patients’ finger joints were acquired, including metacarpophalangeal joints (MCPs), proximal interphalangeal joints (PIPs) and distal interphalangeal joints (DIPs), and each of them was interpolated on a Cartesian grid of 1083 × 277 × 116 samples, with spacings of 0.0277 mm, 0.0902 mm, and 0.1487 mm along the axial, lateral, and elevation directions, respectively. Further details on the acquisition setup are available in [39]. The synovial region was annotated in random 2D slices across the elevation direction of the 3D US images by manually delineating the hypoechoic area above the finger bones using ITK-SNAP software (version 4.0.2) [40].

### 2.2. Data Pre-Processing

**Figure 2:**
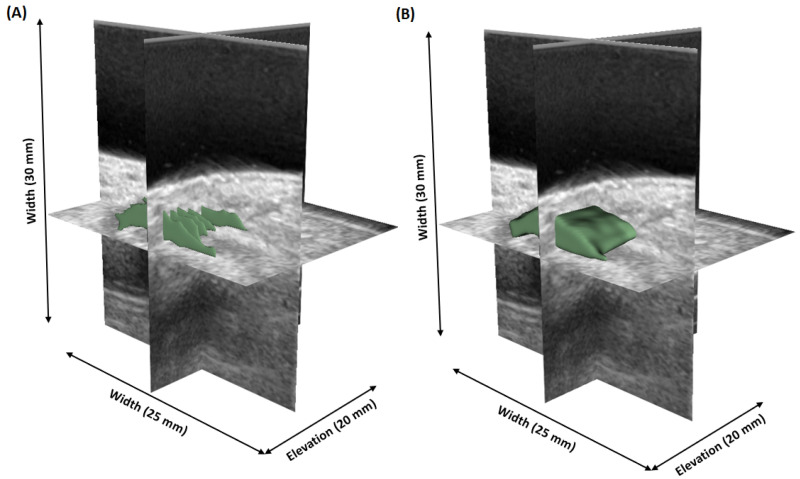
An example of a 3D manual sparse annotation (**A**) and its corresponding generated 3D dense annotation (**B**).

To generate dense 3D synovium annotations from sparse manual annotations, we utilized our previously developed DL-based semi-automated sparse-to-dense annotation model [41]. This approach involved training a 2D UNet++ [42] model for slice-level segmentation and interpolating the expert 2D sparse annotations from 2D US slices. This sparse-to-dense annotation generation methodology has been qualitatively and quantitatively evaluated, and it demonstrated the capability to provide accurate dense 3D annotations, enhancing the continuity and consistency of the segmentation across all dimensions. Figure 2 shows an example of the 3D sparse-to-dense annotation, demonstrating that the dense 3D masks generated from the sparse annotations maintained consistent and continuous shapes. To further evaluate the reliability of the 3D annotations, an expert radiologist reviewed the generated masks across each consecutive slice within each US 3D volume and corroborated that the marked regions accurately depict the synovium region. The 3D US volumes were centrally cropped in a sagittal view to focus on the finger joint region, and then resized to 256×256×64. Due to the limited availability of annotated 3D US images, data augmentation was crucial. To artificially enhance the size and diversity of training dataset, various offline data augmentation techniques from TorchIO [43], a Python library designed for medical imaging augmentation, were applied on the 3D US images and the corresponding dense 3D annotations. These techniques included horizontal/vertical flip, random rotation, random brightness/contrast adjustment, random Gaussian blur, and random noise.

### 2.3. Network Architectures

In this section, we explore and compare six network architectures for synovium segmentation in 3D US images of finger joints: 3D UNet, which extends 2D capabilities to handle volumetric data; DAF3D, which enhances accuracy with deep attention mechanisms; Swin UNETR, which integrates UNet with Swin Transformers to effectively capture local and long-range dependencies; UNETR++, which refines feature representations by incorporating efficient paired attention modules in both the encoder and decoder; TransUNet, which incorporates transformer-based encoders into the UNet architecture to capture global context; and our proposed SwinDAF3D, which combines Swin FPN with a deep attention mechanism for optimized feature extraction in synovium segmentation task.

#### 2.3.1. 3D UNet

**Figure 3:**
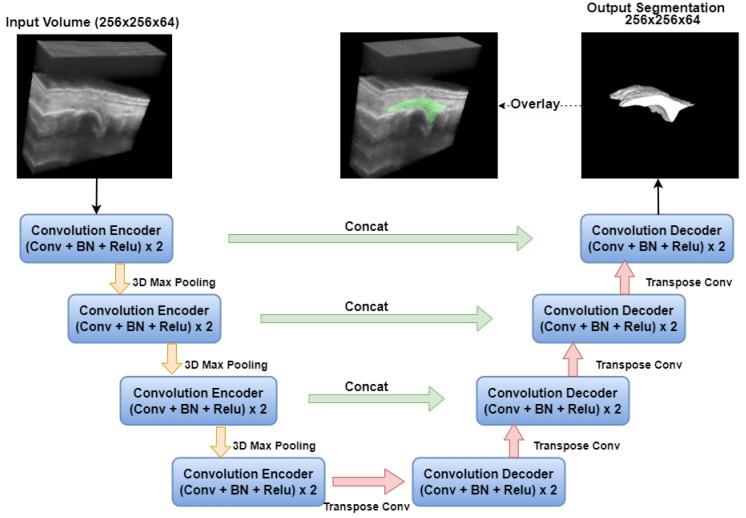
Three-dimensional UNet network architecture for synovium segmentation in 3D US images of finger joints. Conv: convolution; BN: batch normalization; ReLu: ReLu activation.

The implemented 3D UNet [26] model, depicted in Figure 3, consists of two main components: a downsampling path (encoder) and an upsampling path (decoder), both conducting operations in 3D. The downsampling path includes four convolutional encoder blocks, each comprising two sets of 3D convolution layers with a kernel size of 3×3×3. Following each convolution, there is batch normalization and a rectified linear unit (ReLU) [44] activation function. Downsampling is achieved through 3D max pooling layers with a window size of 2×2×2 and strides of two in each dimension. To prevent bottlenecks and ensure an sufficient number of feature channels, the channel count is doubled before each downsampling step. In the upsampling path, there are also four convolutional decoder blocks. Each layer in this path starts with a 3D transposed convolution using a 2×2×2 kernel and strides of two in each dimension. Subsequently, two 3D convolutional layers with 3×3×3 kernels are applied, each followed by a ReLU activation. Skip connections are employed to link corresponding layers from the downsampling path to the upsampling path.

The 3D UNet’s relatively simple architecture has limitations when it comes to capturing complex and ambiguous structures in 3D US images of finger joints. Additionally, its convolutional operations, which excel at capturing localized 3D information, may not effectively capture global, long-range spatial relationships.

#### 2.3.2. DAF3D

**Figure 4:**
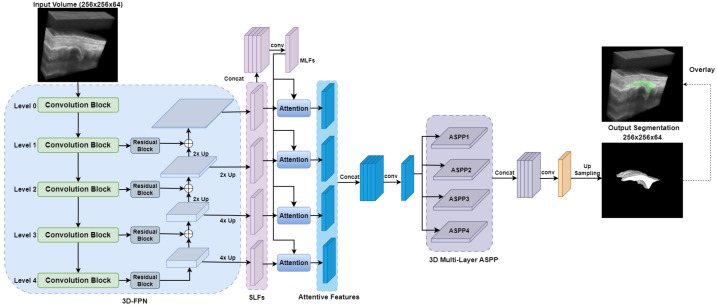
DAF3D network architecture for synovium segmentation in 3D US images of finger joints. FPN: Feature Pyramid Network; SLF: single-layer features; MLF: multi-layer features; ASPP: Atrous spatial pyramid pooling.

The DAF3D [35] network architecture is illustrated in Figure 4. Originally proposed for the 3D transrectal US prostate segmentation task, it is built around a four-level 3D feature pyramid network (FPN) with a ResNeXt [36] backbone enhanced with dilated convolutions to capture multi-scale contextual information. This architecture incorporates Multi-Layer Features (MLFs) and an innovative deep attention mechanism, which computes attention weights for these MLF, refining Single-Layer Features (SLF) through element-wise multiplication.

The attention module consists of three convolutional layers with group normalization and parametric rectified linear unit (PReLU) [45] activation, which collectively produce an attention map that enhances feature representation. Additionally, the 3D Atrous Spatial Pyramid Pooling (ASPP) [46] module further processes these features to address scale variability in synovium shapes.

The primary advantage of DAF3D lies in its ability to integrate multi-level features via attention mechanisms, enhancing both detailed and semantic features at various network depths. This results in more accurate and robust segmentation, effectively handling challenges such as ambiguous boundaries and inhomogeneous intensity distributions, which are common in 3D US imaging.

#### 2.3.3. Swin UNETR

**Figure 5:**
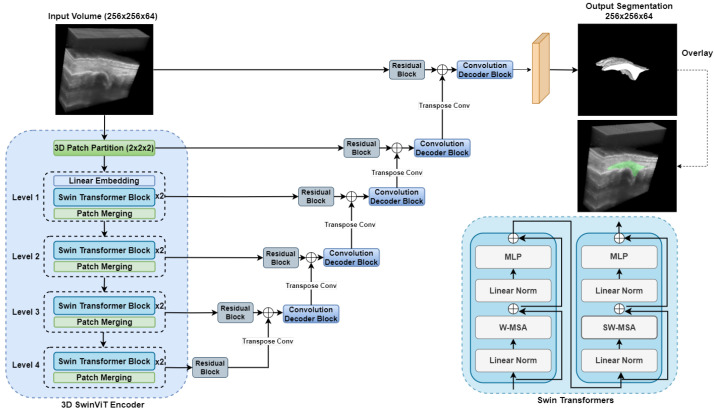
Swin UNETR network architecture for synovium segmentation in 3D US images of finger joints. MLP: Multi-layer Perceptron; W-MSA: window-based Multi-Head Self-Attention; SW-MSA is shifted window-based Multi-Head Self-Attention.

Figure 5 illustrates the network architecture of the Swin UNETR model [31], a hybrid design that integrates Swin Transformers as the encoder and a fully convolutional network as the decoder, similar to the 3D UNet [26]. It leverages the capabilities of transformers in capturing long-range dependencies and the efficiency of CNNs in extracting local features. The input to Swin UNETR is a 3D US image of finger joint $X\in R^{H\times W\times D}$, where H,W,D are spatial dimensions of 3D US image. The input image is divided into non-overlapping patches of size 2×2×2, and each patch is flattened and projected into an embedding space of dimension *C*, with *C* set to 48 in the implementation. The encoder comprises four stages, each containing multiple Swin Transformer blocks that compute self-attention within local windows with size 7×7×7. Windows are also shifted between layers to enable cross-window connections. Within the Swin Transformer blocks, the output of the subsequent layers *l* and l+1 (denoted as $z^{l}$ and $z^{l+1}$) are calculated as follows:

(1) $$\begin{array}{c} {\hat{z}}^{l}=W-\mathrm{MSA}\left(\mathrm{LN}\left(z^{l-1}\right)\right)+z^{l-1}\\ z^{l}=\mathrm{MLP}\left(\mathrm{LN}\left({\hat{z}}^{l}\right)\right)+{\hat{z}}^{l}\\ {\hat{z}}^{l+1}=\mathrm{SW}-\mathrm{MSA}\left(\mathrm{LN}\left(z^{l}\right)\right)+z^{l}\\ z^{l+1}=\mathrm{MLP}\left(\mathrm{LN}\left({\hat{z}}^{l+1}\right)\right)+{\hat{z}}^{l+1}. \end{array}$$

Here, W-MSA and SW-MSA denote window-based multi-head self-attention using regular and shifted window partitioning configurations, respectively; ${\hat{z}}^{l}$ and ${\hat{z}}^{l+1}$ denote the outputs of W-MSA and SW-MSA; MLP and LN denote Multi-Layer Perceptron and layer normalization, respectively. Finally, the self-attention is computed as follows:

(2) $$\mathrm{Attention}\left(Q,K,V\right)=\mathrm{Softmax}\left(\frac{QK^{⊤}}{\sqrt{d}}\right)V.$$

In which Q,K,V are the queries, keys, and values, respectively, and *d* represents the dimension of the queries and keys [47].

At the end of each level, a patch merging layer reduces the spatial dimensions of the feature maps by a factor of 2 while increasing the channel dimension. Features extracted at different resolutions from the encoder are passed to the decoder via skip connections. The decoder comprises five convolutional residual blocks, each consisting of two 3×3×3 convolutional layers with instance normalization. These blocks refine the feature representations and generate the synovium segmentation.

The Swin UNETR architecture effectively combines the hierarchical feature extraction capabilities of Swin Transformers with the efficient local feature processing of convolutional networks, capturing both global and local context comprehensively.

#### 2.3.4. UNETR++

**Figure 6:**
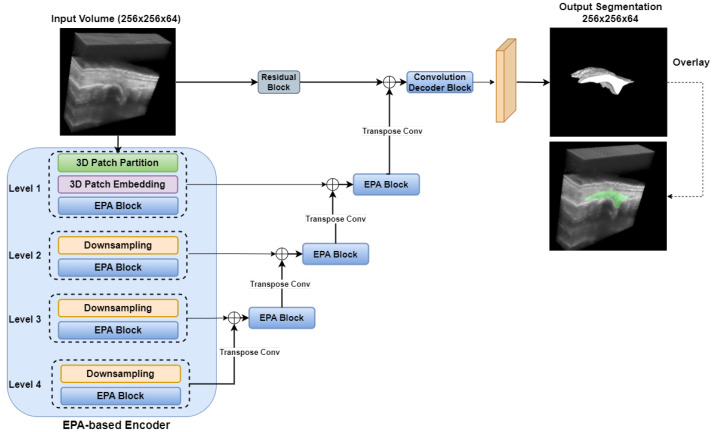
UNETR++ network architecture for synovium segmentation in 3D US images of finger joints. EPA: Efficient Paired-Attention.

The network architecture of the UNETR++ model is depicted in Figure 6. UNETR++ [37] builds on the original UNETR [48] framework by integrating a novel efficient paired-attention (EPA) block that simultaneously captures spatial and channel dependencies, thereby enhancing segmentation accuracy while reducing computational overhead [37]. The model employs an encoder–decoder architecture in which EPA blocks are embedded at every stage of both the encoder and the decoder. The key innovation of UNETR++ lies in its dual-branch EPA module, which comprises two complementary attention mechanisms with shared query and key mappings. This design enables the network to focus on both the spatial layout and the channel-wise relationships of the input data.

In the spatial branch, the EPA block reduces the complexity of traditional self-attention from quadratic to linear by projecting the keys and values into a lower-dimensional space. For a given input feature map, shared queries ($Q_{shared}$) and keys are computed, and the keys are further projected to a lower-dimensional space ($K_{proj}$). The spatial attention map is then obtained via a weighted sum of the projected values (${\tilde{V}}_{\mathrm{spatial}}$), as defined by [37]:

(3) $${\hat{X}}_{s}=\mathrm{Softmax}\left(\frac{Q_{shared}\;K_{proj}^{⊤}}{\sqrt{d}}\right)\cdot {\tilde{V}}_{spatial},$$

where *d* is the dimensionality of the queries and keys.

Concurrently, the channel branch addresses the interdependencies between feature channels. Using the same shared queries and keys, the channel attention is computed by creating a dot product in the channel dimension, which emphasizes the correlations among different feature maps [37]:

(4) $${\hat{X}}_{c}=V_{channel}\cdot \mathrm{Softmax}\;\left(\frac{Q_{shared}^{⊤}\;K_{shared}}{\sqrt{d}}\right),$$

with $V_{channel}$ representing the channel-specific value projection.

The outputs of the spatial and channel attention modules are subsequently fused through a series of convolutional layers to generate an enriched feature representation. This fusion not only integrates complementary information from both branches but also refines the feature maps for improved segmentation performance.

Overall, the UNETR++—with its EPA attention mechanism and hierarchical structure—demonstrates significant potential for improving volumetric segmentation tasks by learning enriched inter-dependent spatial and channel features.

#### 2.3.5. TransUNet

**Figure 7:**
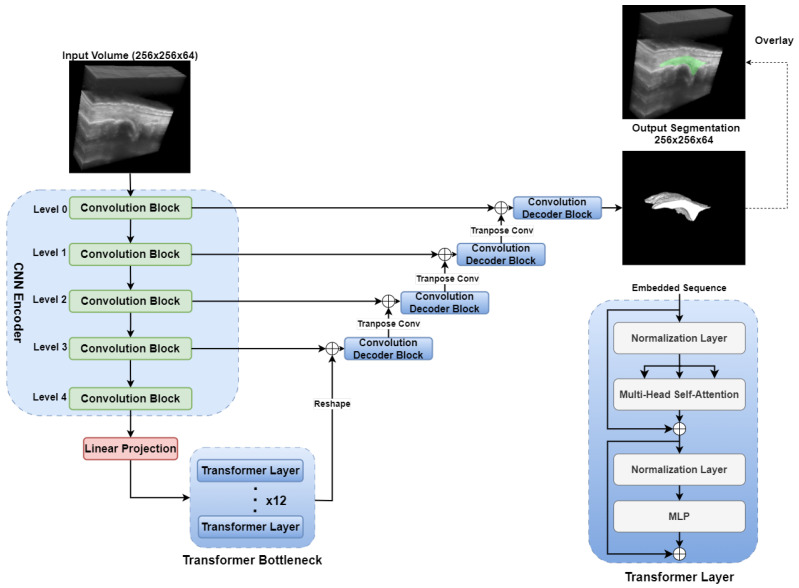
TransUNet network architecture for synovium segmentation in 3D US images of finger joints. MLP: Multi-layer Perceptron.

The network architecture of the TransUNet model is depicted in Figure 7. We implemented a 3D variant of the original TransUNet architecture proposed by Chen et al. [38] for medical image segmentation tasks. The TransUNet architecture integrates the strengths of the UNet’s ability to capture detailed local features with the Transformer’s self-attention mechanism for global context modeling. The architecture consists of three primary components: an encoder, a Transformer-based bottleneck, and a decoder enhanced with skip connections.

The model initially employs an encoder path structured similarly to a traditional 3D UNet, leveraging successive convolutional blocks and downsampling operations to extract hierarchical, high-dimensional features from volumetric input data. Each convolutional block consists of two 3D convolutional layers, each followed by instance normalization and ReLU activation. At the bottleneck, CNN-extracted features are reshaped into sequential token representations for processing by a 12-layer Transformer encoder. Positional embeddings are incorporated to preserve spatial information.

A series of Transformer encoder layers then apply multi-head self-attention mechanisms, enabling the model to capture long-range dependencies and global contextual information beyond the local receptive fields of conventional CNN-based encoders. The decoder adopts a CNN-based approach, using transposed convolutions for upsampling. Skip connections from corresponding encoder layers are concatenated at each decoding stage to integrate high-resolution features. Following each concatenation, convolutional blocks consisting of two sequential 3D convolutions, instance normalization, and ReLU activation further refine the fused features.

By effectively capturing long-range dependencies and global context, TransUNet can enhance segmentation accuracy. Its adaptive Transformer-based architecture offers flexibility across diverse medical segmentation tasks.

#### 2.3.6. SwinDAF3D

**Figure 8:**
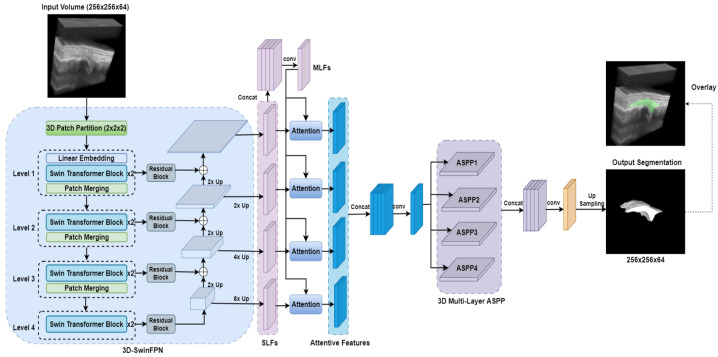
SwinDAF3D network architecture for synovium segmentation in 3D US images of finger joints. FPN: Feature Pyramid Network; SLF: single-layer features; MLF: multi-layer features; ASPP: Atrous spatial pyramid pooling.

Figure 8 shows the network architecture of our proposed SwinDAF3D model, which integrates the advantages of Swin UNETR [31] and DAF3D [35], aiming to leverage the strengths of both models for improved performance. The same Swin Transformer encoder from Swin UNETR is utilized to construct a four-level Swin FPN, capturing hierarchical multi-scale contextual information from 3D US images. Multi-scale features from different levels of the Swin FPN are extracted and concatenated to form the MLF.

Attention weights are then computed for the MLF to enhance relevant features and suppress irrelevant ones. The attention weight computation is defined as follows:

(5) $$A_{i}=\sigma \left(f_{a}\left(\left(F_{i},F_{MLF}\right);\theta \right)\right),$$

where $A_{i}$ represents the computed attention weights at level *i*, $F_{i}$ denotes the SLF extracted from level *i* of Swin FPN, $F_{MLF}$ denotes the MLF, θ represents the parameters learned by $f_{a}$, which contains three 3D convolutional layers, and σ is the Sigmoid activation function. The weighted MLF denoted as $F_{MLF}^{\prime }$ is computed by element-wise multiplication of $F_{MLF}$ and $A_{i}$:

(6) $$F_{MLF}^{\prime }=A_{i}⊙F_{MLF}.$$

Finally, the attentive features are computed by merging $F_{MLF}^{\prime }$ with corresponding $F_{i}$ by applying two 3×3×3 and one 1×1×1 convolutional layers. This process refines the layer-wise SLF and produces the final attentive features for the given Swin FPN level. The computed attentive features are then concatenated and go through 3D multi-layer ASPP and convolution blocks to generate the synovium segmentation.

SwinDAF3D combines Swin UNETR’s hierarchical and long-range dependency modeling capabilities with DAF3D’s feature refinement strengths via a deep attention mechanism. This integration provides an effective approach to capturing and enhancing multi-scale US features.

### 2.4. Design of Ablation Study

To rigorously assess the contribution of each architectural component in our proposed SwinDAF3D model, we conducted a comprehensive ablation study comparing it against five established baseline models (summarized in Table 1). Each baseline was chosen to isolate and evaluate the impact of the backbone type, attention mechanism, and the level at which transformer components are integrated. The objectives of each model comparison are described below:

- 3D UNet: Serves as a standard baseline CNN model for evaluating segmentation performance.
- DAF3D: Assesses the performance gains achieved by employing a hierarchical Swin Transformers backbone as opposed to a conventional CNN-based backbone like ResNeXt.
- Swin UNETR: Assesses the incremental benefits obtained by integrating deep attention mechanisms into the hierarchical Swin Transformers backbone.
- UNETR++: Compares the relative effectiveness of deep attention mechanisms against efficient paired attention (EPA) modules within a hierarchical transformer framework.
- TransUNet: Assesses the advantages of fully integrating hierarchical Swin Transformers and deep attention mechanisms compared to architectures that use transformers only at the bottleneck stage.

In this study, we performed six-fold cross-validation with a dataset of clinically confirmed cases of RA (*n* = 72). Additionally, we tested the models on an independent test set, excluded from the training and validation sets, with clinically confirmed cases of RA (*n* = 30).

### 2.5. Model Training

For all the networks, including 3D UNet, DAF3D, Swin UNETR, UNETR++, TransUNet, and proposed SwinDAF3D, Dice loss and binary cross-entropy (BCE) loss are computed at the output of each network. The Dice loss is defined as follows:

(7) $$L_{Dice}=1-\frac{2\sum_{i=1}^{N}P_{i}G_{i}}{\sum_{i=1}^{N}P_{i}^{2}+\sum_{i=1}^{N}G_{i}^{2}}$$

where *N* is the number of voxels in the input 3D US volumes, $P_{i}$ is the predicted probability for voxel *i*, and $G_{i}$ is the ground truth label for voxel *i*. The binary cross-entropy loss is defined as follows:

(8) $$L_{BCE}=\sum_{i=1}^{N}G_{i}\log\left(P_{i}\right)+\left(1-G_{i}\right)\log\left(1-P_{i}\right)$$

For the 3D UNet, Swin UNETR, UNETR++, and TransUNet networks, which do not have deep attention mechanisms, the total loss denoted as $L_{out}$ is simply the sum of the Dice loss and the BCE loss at the output level of each network:

(9) $$L_{out}=L_{out,Dice}+L_{out,BCE}$$

For DAF3D and SwinDA3D networks, which incorporate deep attention mechanisms, additional Dice loss and BCE loss are also computed for each SLF and output of each attention module:

(10) $$\begin{array}{c} L_{slf}=L_{slf,Dice}+L_{slf,BCE}\\ L_{attn}=L_{attn,Dice}+L_{attn,BCE} \end{array}$$

The total loss, denoted as $L_{total}$, for both DAF3D and the SwinDAF3D network is the sum of the loss across all layers, including the refined layers after applying the attention mechanisms and the output layer. It is defined as follows:

(11) $$L_{\mathrm{total}}=\sum_{i=1}^{N}w_{i}L_{i,slf}+\sum_{j=1}^{M}w_{j}L_{j,attn}+L_{out}$$

Here, N = 4 and M = 4 represent the number of layers before and after applying the attention modules, respectively. $w_{i}$ and $w_{j}$ are the weights assigned to each layer’s loss. We use the same weighting as defined in [35], which weights ($w_{i}$ = 1, 2, 3, 4, $w_{j}$ = 1, 2, 3, 4) empirically as (0.4, 0.5, 0.7, 0.8, 0.4, 0.5, 0.7, 0.8).

All networks in this work were implemented based on PyTorch [49] and MONAI [50], and they were trained on a single NVIDIA Tesla V100 GPU. We used the loss function defined in Equation (9) for 3D UNET, Swin UNETR, UNETR++, and TranUNet, and the loss function defined in Equation (11) for DAF3D and SwinDAF3D. We adopted the Adam optimizer [51] with a learning rate of 0.0001 and a weight decay of 0.0001. All models were trained for 50 epochs with a batch size of one. To enhance the robustness and generalizability of the model and to artificially expand the training dataset’s size and diversity, we applied extensive data augmentation strategies on the 3D US images, including random flip, random rotation, random brightness/contrast, random Gaussian blur, and random noise using the TorchIO library [43]. These transforms are intended to simulate different positioning and angles of the ultrasound probe, as well as varying settings of dynamic range, gain, and transmission power. Regularization was implicitly managed through weight decay, and performance was monitored using both BCE loss and Dice loss metrics. The best-performing model was selected based on the highest Dice coefficient achieved on the validation set.

### 2.6. Model Performance Assessment

The performance metrics for evaluating all networks include the Dice score (DSC), intersection over union (IoU), and the Surface Dice Score (SDSC). The Dice score measures the similarity between the predicted segmentation and the ground truth. It quantifies similarity as follows:

(12) $$DSC=\frac{2\times |A\cap B|}{\left|A|+|B\right|}$$

where *A* represents the set of predicted voxels and *B* represents the set of ground truth voxels. A higher Dice score indicates better overlap between the predicted and ground truth segmentation. IoU measures the ratio of the intersection of the predicted voxels and the ground truth to their union, and it is calculated as follows:

(13) $$IoU=\frac{\left|A\cap B\right|}{\left|A\cup B\right|}$$

IoU gives a straightforward measure of how much the predicted and actual segmentation overlap, with values closer to 1 indicating better segmentation accuracy.

In addition to volumetric-based metrics, the SDSC evaluates segmentation performance at the surface level, which is particularly important for assessing boundary accuracy. The surface of both the predicted segmentation $S_{p}$ and the ground truth segmentation $S_{g}$ is extracted using morphological operations such as binary erosion. The SDSC is then computed as follows:

(14) $$SDSC=\frac{2\;\times \;|S_{p}\cap S_{g}|}{\left|S_{p}|+|S_{g}\right|}$$

where $|S_{p}\cap S_{g}|$ denotes the number of surface voxels from $S_{p}$ that lie within a defined distance *d* of $S_{g}$, and vice versa. In this formulation, the tolerance distance is set to d = 1.

In the cross-validation study, a Wilcoxon signed-rank test with Bonferroni correction was performed to evaluate the differences in DSC, IoU and SDSC values across the six validation folds for each model, determining whether these differences were statistically significant. The Wilcoxon signed-rank test is a non-parametric method that compares paired samples, while the Bonferroni correction adjusts the *p*-values to account for multiple comparisons.

## 3. Results

**Figure 9:**
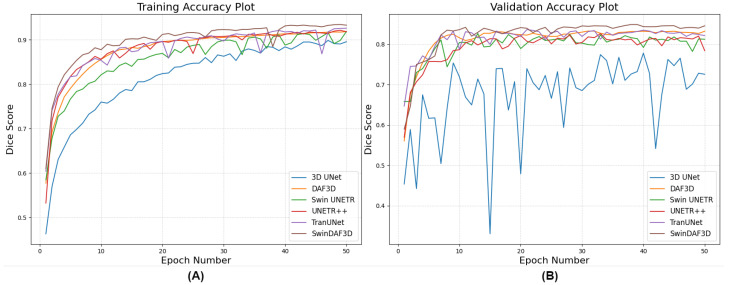
Training (**A**) and validation (**B**) Dice score accuracy curves (one-fold) over 50 epochs for all models in the ablation study: 3D UNet, DAF3D, Swin UNETR, UNETR++, TransUNet, and SwinDAF3D.

Figure 9 illustrates the training and validation Dice score accuracy curves of a single fold over 50 epochs for all networks. The results indicate that the 3D UNet has the lowest Dice score values for both training and validation, highlighting the limitation of CNNs in capturing global, long-range spatial relationships in complex 3D US images of finger joints. DAF3D, Swin UNETR, UNETR++, and TransUNet exhibit similar Dice scores, but the curve for DAF3D is more stable. This stability suggests that the dataset size may be a limitation for Transformer-based models, which typically require larger datasets for optimal training. It also indicates that the use of deep attention in DAF3D aids feature refinement, improving the model’s robustness. Our proposed SwinDAF3D network achieved the highest Dice scores for both training and validation, demonstrating the effectiveness of combining Swin FPN with a deep attention mechanism for capturing attentive hierarchical multi-scale contextual information.

**Figure 10:**
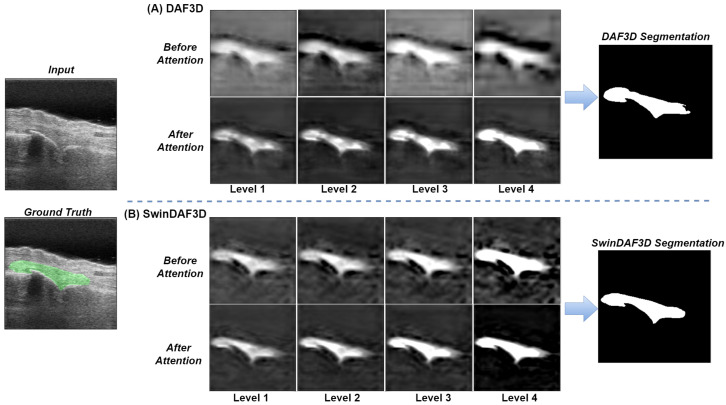
Example feature map visualizations demonstrating the efficacy of feature extraction by integrating Swin transformers feature pyramid (Swin FPN) with attention modules. (**A**) illustrates the DA3D single-layer features (SLFs) from levels 1 to 4 before and after applying attention modules, along with the final segmentation output. (**B**) shows the SwinDAF3D SLFs from levels 1 to 4 before and after applying attention modules, alongside the final segmentation output. We can observe that Swin FPN’s feature extraction provides superior feature representation at each level, and the attention module effectively refines these features.

In the feature map visualizations presented in Figure 10, we demonstrate the efficacy of integrating Swin FPN with a deep attention mechanism for feature extraction. Figure 10A illustrates the DA3D SLFs across levels 1 to 4, both before and after applying attention modules, culminating in the final segmentation output. Similarly, Figure 10B displays the SwinDAF3D SLFs from levels 1 to 4, also before and after applying attention modules, along with the final segmentation output. The results clearly indicate that Swin FPN significantly enhances feature representation across all levels comparing to ResNeXt FPN, providing a more detailed and granular feature set. Furthermore, the attention modules are effective in refining these features, leading to improved segmentation accuracy. This demonstrates the robustness of the combined Swin FPN and attention module approach in extracting and refining hierarchical features for better segmentation performance.

Performance metrics for the six-fold cross-validation (*n* = 72) and independent test (*n* = 30) of all networks are summarized in Table 2. We used the same Swin Transformer encoder configuration as proposed in SwinUNETR [31], with a feature size of 48 and window size of 7 × 7 × 7. During cross-validation, the highest evaluation DSC and IoU were selected for each fold. For each model type, the fold with the highest DSC and IoU was used to evaluate the independent test set. Overall, the proposed SwinDAF3D model achieved the highest performance, with a DSC of 0.838 ± 0.013, an IoU of 0.719 ± 0.019, and a SDSC of 0.852 ± 0.020 for cross-validation, and a DSC of 0.825, an IoU of 0.692, and a SDSC of 0.832 for the independent test set. The SwinDAF3D outperforms the 3D UNet (*p* < 0.03, *n* = 6), DAF3D (*p* < 0.05, *n* = 6), Swin UNETR (*p* < 0.05, *n* = 6), UNETR++ (*p* < 0.05, *n* = 6), and TransUNet (*p* < 0.05, *n* = 6). Additionally, we conducted a post hoc power analysis based on the DSC metrics of the SwinDAF3D model. Specifically, the effect size was estimated from the observed differences in DSC between SwinDAF3D and the baseline model with highest mean DSC (TransUNet). Using the Wilcoxon signed-rank test at an alpha level of 0.05, the analysis indicated that our cross-validation study achieved a power of approximately 85% in detecting the reported significant improvements.

In addition to the ablation studies comparing different network architectures, we conducted a parameter sensitivity analysis for the proposed SwinDAF3D model. As summarized in Table 3, varying the feature size while keeping the window size constant at seven revealed that the model’s performance improves with larger feature sizes, reaching an optimal performance at a feature size of 48 (DSC: 0.825, IoU: 0.692, SDSC: 0.832), with marginal gains observed at a feature size of 60. Similarly, experiments varying the local window size with a fixed feature size of 48 indicated that a window size of seven achieves the best performance, compared to smaller (three and five) or larger (nine) window sizes. These results demonstrate the robustness of the SwinDAF3D architecture and validate the selection of a feature size of 48 and a window size of seven as the optimal configuration for synovium segmentation in 3D US images.

**Figure 11:**
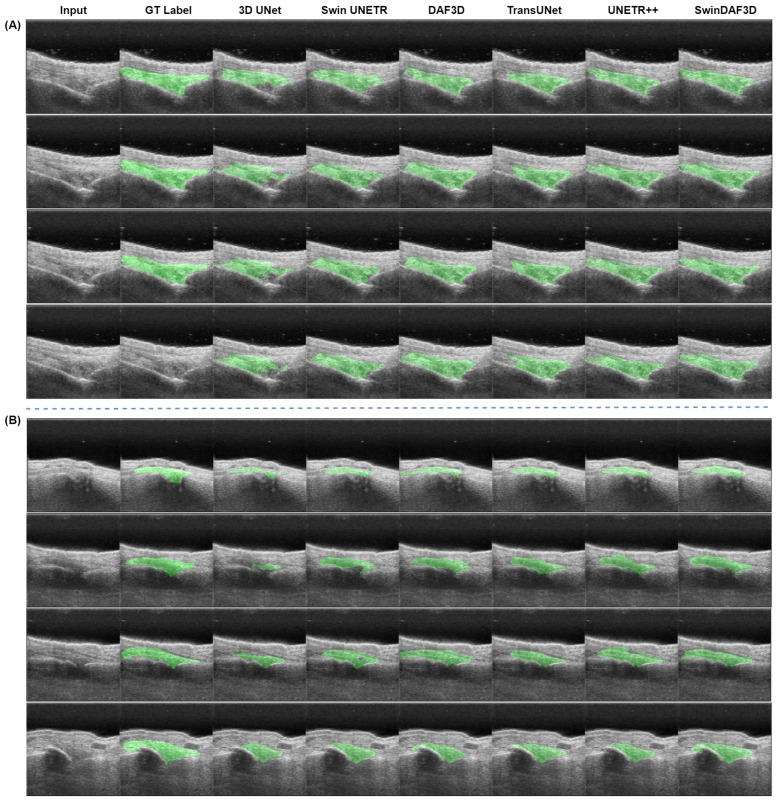
Test set sample segmentation results from the 3D UNet, Swin UNETR, DAF3D, TransUNet, UNETR++, and SwinDAF3D models are compared against their corresponding ground truth annotations, with both segmentation and ground truth masks overlaid in green. (**A**) Ultrasound images with higher quality and larger synovium size; (**B**) challenging ultrasound images featuring a larger shadow, reduced contrast, and a smaller synovium.

Figure 11 presents example segmentation results of the 3D UNet, Swin UNETR, DAF3D, TransUNet, UNETR++, and SwinDAF3D models compared to their corresponding ground truth annotations. The 3D UNet model demonstrates the poorest performance in synovium segmentation, often failing to capture the synovium accurately. DAF3D, Swin UNETR, UNETR++, and TransUNet models show improved segmentation but exhibit some missing regions and holes along the synovium boundaries and within the synovium itself. In contrast, the SwinDAF3D model produces more detailed and complete segmentations, accurately representing the granular and comprehensive shape of the synovium.

All computation performance metrics were measured on a NVIDIA Tesla V100 GPU. Table 4 compares the computational cost (GFLOPs), memory usage (MB), and inference time (s) of the models evaluated in the ablation study, including 3D UNet, DAF3D, Swin UNETR, UNETR++, TransUNet, and SwinDAF3D. The UNETR++ model achieves the lowest computational burden, requiring only 262 GFLOPs, using 1165 MB of memory, and achieving an inference time of 0.085 s—substantially outperforming the simple CNN-based 3D UNet model, which operates at 1006 GFLOPs, 2536 MB, and 0.175 s. In comparison, DAF3D and TransUNet present moderate computational demands with 1263 and 1182 GFLOPs, memory usage of 1958 MB and 4125 MB, and inference times of 0.201 s and 0.189 s, respectively. Swin transformer-based models, particularly Swin UNETR and SwinDAF3D, incur higher computational cost (1522 GFLOPs and 1730 GFLOPs), increased memory consumption (7227 MB and 7597 MB), and longer inference times (0.343 s and 0.375 s), reflecting their increased complexity due to hierarchical Swin Transformers architecture.

## 4. Discussion

In this work, we presented the novel SwinDAF3D architecture, which demonstrated the feasibility to provide highly accurate, automatic segmentation of the synovium region in 3D US images, showing promise in establishing standardized clinical workflows for RA diagnosis and assessment. Our comparative analysis showed that the proposed architecture provided more accurate segmentation in terms of DSC and IoU, and SDSC as compared to baseline models: 3D UNet, DAF3D, Swin UNETR, UNETR++, and TransUNet. The improved performance demonstrated that the innovative integration of Swin FPN with a deep attention mechanism, enhances the model’s capability to capture rich, multi-scale contextual information and long-range dependencies.

The results from our six-fold cross-validation and independent test set evaluations indicate significant performance variations across these models, providing insights into their respective strengths and limitations. The 3D UNet consistently demonstrated the lowest DSC, IoU, and SDSC values, highlighting its limitations in accurately capturing the complex and intricate structures of the synovium in 3D US images. This observation is consistent with previous studies [52,53,54], suggesting that traditional CNNs may not effectively capture global context and long-range dependencies in 3D data, leading to suboptimal segmentation outcomes.

DAF3D, Swin UNETR, UNETR++, and TransUNet models showed better performance compared to 3D UNet. The DAF3D model, which incorporates a deep attention mechanism, exhibited a more stable Dice score curve during training and validation, indicating that DAF3D effectively refines multi-layer features, enhancing its robustness and segmentation accuracy. Conversely, Swin UNETR, UNETR++, and TransUNet, despite their competitive performance, displayed sensitivity to dataset size, likely due to the requirement for larger training datasets to train transformer-based architectures.

The proposed SwinDAF3D model achieved the highest DSC, IoU and SDSC metrics, outperforming other baseline models in the study. The integration of Swin FPN with a deep attention mechanism in SwinDAF3D effectively captures hierarchical multi-scale contextual information, leading to more accurate and comprehensive segmentation of the synovium. This model’s encouraging performance demonstrates the importance of combining advanced transformer-based architectures with deep attention mechanisms to enhance segmentation performance in 3D medical imaging tasks. The segmentation results illustrated in Figure 11 further substantiate our quantitative findings. The SwinDAF3D model not only achieved the highest accuracy but also produced more granular and complete synovium segmentation. This level of detail is crucial for clinical applications where precise delineation of anatomical structures is essential.

While the results of this study are promising, several limitations should be noted. The dataset used for training and validation was relatively small, potentially rendering the model prone to overfitting and limiting its generalization capabilities. To artificially increase the dataset’s size and variability, we applied random augmentation transforms, including random rotation, brightness, contrast, and noise addition, while weight decay was used for regularization of the training process. Additionally, we performed six-fold cross-validation and testing on an independent set to enhance the robustness of the model’s performance assessment. Ongoing efforts involve acquiring larger and more diverse datasets from different operators and varying US acquisition settings to further improve the model’s ability to handle variability in real-world clinical data.

Another matter warranting further exploration is the use of semi-automated generation of dense 3D annotations. Previous studies have mostly focused on training 2D segmentation models for synovium segmentation using fully manual 2D expert annotations as ground truth [55,56,57]. Due to the challenging nature of manually annotating 3D US data, we employed the DL-based methodology previously proposed and validated in [41], which utilizes manual 2D expert annotations to generate 3D dense annotations. To further verify the reliability of the 3D annotations, an expert radiologist confirmed that the generated annotations accurately depict the synovial region. Additional validation of the sparse-to-dense annotation methodology could further improve the robustness of this study, where the generated 3D annotations would be compared against ground truth labels from multiple experts in terms of inter-rater segmentation accuracy metrics.

In a clinical setting, the proposed 3D segmentation model is designed to operate locally, on a dedicated GPU within the US acquisition setup. The model will process B-mode US images immediately after acquisition. Another potential application involves integrating the model with PACS systems using standard protocols like DICOM to further enhance clinical workflows. This integration ensures that segmented images are readily accessible to radiologists and other healthcare professionals, facilitating seamless storage, retrieval, and sharing of imaging data. Moreover, the model’s near real-time inference capability would allow for the immediate processing of US images during examinations. This can provide clinicians with instant feedback, aiding in quicker decision-making.

Finally, the imaging setup used in this study enables simultaneous and co-localized acquisition of PA images. As the next step in our research, we will apply the segmentation model in 3D B-mode US images to identify regions for obtaining PD and PA measurements. Such measurements will be used to quantify hemoglobin levels in the synovial tissue, which is a crucial biomarker for RA and has the potential to significantly enhance the sensitivity of RA detection.

## 5. Conclusions

In conclusion, a novel deep learning architecture, SwinDAF3D, was presented. It combines Swin Transformers with a deep attention mechanism, enabling an enhanced capability to capture comprehensive, multiscale contextual information in 3D US images. The proposed model provided superior performance in segmenting complex synovium structures in 3D US images, as compared to four baseline DL architectures for 3D segmentation. This advancement shows great promise for enabling more efficient and standardized clinical workflows for RA diagnosis using US imaging.

## Figures and Tables

**Table 1:** Comparison of model architectures in the ablation study. This table provides a comprehensive overview of each model’s key components, including the the use of CNN backbones, transformer backbones, attention mechanisms, and the specific levels of transformer integration.

Model Name	CNN Backbone	Transformer Backbone	Attention Mechanism	Transformer Integration Level
3D UNet [26]	✓	–	–	–
DAF3D [35]	✓	–	✓(Deep Attention)	–
Swin UNETR [31]	–	✓(Swin)	–	Encoder
UNETR++ [37]	–	✓	✓(EPA)	Encoder & Decoder
TransUNet [38]	✓	✓	–	Bottleneck
SwinDAF3D	–	✓(Swin)	✓(Deep Attention)	Encoder

**Table 2:** Segmentation performance statistics of six-fold cross-validation and independent test set in terms of dice score (DSC), intersection over union (IoU), and surface Dice score (SDSC) for all models in the ablation study: 3D UNet, DAF3D, Swin UNETR, UNETR++, TransUNet, and SwinDAF3D.

	3D UNet			DAF3D			Swin UNETR			UNETR++			TransUNet			SwinDAF3D		
Metrics	DSC	IoU	SDSC	DSC	IoU	SDSC	DSC	IoU	SDSC	DSC	IoU	SDSC	DSC	IoU	SDSC	DSC	IoU	SDSC
Fold 1	0.723	0.566	0.639	0.835	0.715	0.838	0.829	0.706	0.863	0.833	0.711	0.876	0.842	0.722	0.823	0.854	0.745	0.887
Fold 2	0.747	0.596	0.675	0.814	0.697	0.826	0.768	0.628	0.798	0.791	0.659	0.801	0.817	0.690	0.789	0.815	0.684	0.845
Fold 3	0.784	0.638	0.638	0.801	0.673	0.814	0.802	0.674	0.823	0.808	0.685	0.818	0.805	0.674	0.796	0.841	0.723	0.821
Fold 4	0.713	0.555	0.623	0.833	0.714	0.821	0.835	0.704	0.822	0.801	0.671	0.811	0.807	0.677	0.822	0.832	0.709	0.851
Fold 5	0.721	0.563	0.687	0.788	0.653	0.798	0.831	0.708	0.869	0.821	0.701	0.857	0.824	0.703	0.831	0.849	0.733	0.864
Fold 6	0.763	0.617	0.701	0.811	0.683	0.805	0.785	0.645	0.755	0.807	0.675	0.814	0.812	0.683	0.828	0.835	0.719	0.841
Mean	0.742	0.589	0.661	0.813	0.689	0.817	0.808	0.678	0.822	0.810	0.684	0.829	0.818	0.692	0.815	0.838	0.719	0.852
Std	0.025	0.031	0.029	0.017	0.022	0.013	0.025	0.032	0.039	0.014	0.018	0.027	0.013	0.017	0.016	0.013	0.019	0.020
Test Set	0.691	0.571	0.633	0.768	0.643	0.793	0.788	0.654	0.814	0.779	0.646	0.811	0.781	0.651	0.798	0.825	0.692	0.832

**Table 3:** Segmentation performance metrics on the independent test set for SwinDAF3D with varying feature size and local window size.

Feature Size (Window Size = 7)				Window Size (Feature Size = 48)			
Size	DSC	IoU	SDSC	Size	DSC	IoU	SDSC
24	0.793	0.658	0.801	3	0.778	0.641	0.793
36	0.811	0.676	0.818	5	0.808	0.671	0.831
48	0.825	0.692	0.832	7	0.825	0.692	0.832
60	0.827	0.695	0.833	9	0.824	0.690	0.837

**Table 4:** Comparison of computational cost, memory usage, and inference time for all models in the ablation study: 3D UNet, DAF3D, Swin UNETR, UNETR++, TransUNet and SwinDAF3D.

Model Name	GFLOPs	Memory Usage (MB)	Inference Time (s)
3D UNet	1006	2536	0.175
DAF3D	1263	1958	0.201
Swin UNETR	1522	7227	0.343
UNETR++	262	1165	0.085
TransUNet	1182	4125	0.189
SwinDAF3D	1730	7597	0.375

## Data Availability

Retrospective data were collected at University of Michigan and cannot be shared publicly.
